# Predicting teacher turnover in private universities: a machine learning approach based on 10 years of data and satisfaction factors

**DOI:** 10.3389/fpsyg.2025.1670195

**Published:** 2025-11-06

**Authors:** Wang Jingwen, Liu Yi, Yang Xiaohong

**Affiliations:** 1 General Office of the Party and Government, Xi'an Fanyi University, Xi'an, China; 2 College of Educational Technology, Northwest Normal University, Lanzhou, China

**Keywords:** teacher turnover, private universities, machine learning, exploratory factor analysis, job satisfaction

## Abstract

**Background:**

Teacher turnover poses a significant challenge to the sustainable development of private universities in China. While machine learning (ML) has been increasingly applied to turnover prediction, existing studies often overlook psychological factors and lack longitudinal analysis.

**Methods:**

This study integrates a 10-year longitudinal dataset with satisfaction surveys from a private university in Western China. Exploratory Factor Analysis (EFA) was employed to extract key dimensions influencing turnover. Three ML models—K-Nearest Neighbors (KNN), Naive Bayes (NB), and Backpropagation Neural Network (BPNN)—were constructed and evaluated using accuracy, F1-score, and AUC.

**Results:**

The KNN model achieved the highest predictive performance (accuracy = 83.64%, F1 = 84.16%, AUC = 0.901). The “Compensation, Benefits, and Development” dimension was identified as the most influential factor, accounting for 25.41% of the variance.

**Conclusion:**

This study proposes an “EFA + ML” hybrid approach that enhances feature interpretability and prediction robustness, offering practical insights for human resource management in private higher education institutions.

## 1 Introduction

With the continuous development of China’s higher education system, private universities have become an indispensable part of this landscape, playing a crucial role in promoting educational equity and diversity. According to the “2023 National Education Development Statistical Bulletin” released by the Ministry of Education, there are 789 private universities in China, accounting for 25.67% of the total number of universities. Private universities also enroll 26.34% of the total student population across ordinary, vocational, and junior colleges, and employ 20.13% of all full-time teachers in Chinese universities (Ministry of Education of the People's Republic of China, 2024).

Despite substantial growth in both number and student enrollment, private universities continue to experience difficulties in recruiting and retaining high-quality faculty. This challenge has emerged as a key constraint on the sustainable development of these institutions. Owing to their “non-enterprise” and “extra-system” status, faculty in private universities often experience systemic inequities compared to their public-university counterparts in areas such as political standing, social recognition, and welfare benefits. These disparities not only undermine job satisfaction and loyalty among teachers, but are also associated with higher turnover rates. Studies show that 33.5% of teachers in private universities exhibit turnover intention (Qi, 2023), with actual annual turnover rates reaching as high as 10% in some cases (Bai, 2018). High faculty attrition increases recruitment and training costs, and adversely affects teaching quality, academic development, and long-term institutional planning.

In response to the issue of teacher turnover, researchers in China and abroad have adopted a variety of approaches, including both traditional statistical methods and machine learning techniques. Conventional methods such as descriptive statistics, regression analysis, factor analysis, and mediation analysis have played significant roles in predicting employee turnover. However, their applicability and predictive power are often limited by strict assumptions, such as normality and linearity. In contrast, machine learning methods—such as decision trees, random forests, K-Nearest Neighbors (KNN), and Naive Bayes (NB), Backpropagation Neural Network (BPNN)—can more effectively capture non-linear relationships and complex decision boundaries, thereby enhancing predictive accuracy and efficiency. Nonetheless, existing machine learning studies on teacher turnover have primarily focused on corporate employees and relied heavily on objective data such as salary levels, while paying insufficient attention to psychological traits such as job satisfaction and professional efficacy. Moreover, the absence of feature importance analysis in many studies limits their utility in formulating targeted teacher retention strategies.

Based on the aforementioned research gap, this study proposes two research questions: (1) What are the key dimensions influencing faculty turnover in private universities, and what is the relative importance (predictive weight) of each dimension in predicting turnover? (2) Among the three machine learning algorithms—KNN, NB, and BPNN Neural Network—which model demonstrates superior accuracy and stability in predicting faculty turnover in private universities?

Furthermore, grounded in classical theoretical frameworks such as Herzberg’s Two-Factor Theory and Maslow’s Hierarchy of Needs, this study puts forward three main research hypotheses: H1: Job satisfaction is negatively correlated with turnover intention. H2: The “Compensation, Benefits, and Development” dimension (including hygiene factors such as salary, security, and promotion) has a stronger predictive power on turnover than other dimensions. H3: The KNN model outperforms both the NB and BPNN models in terms of accuracy, F1-score, and AUC value in predicting faculty turnover.

The introduction of a longitudinal tracking design is a key methodological feature of this study. Multiple studies have demonstrated that, compared to cross-sectional data, longitudinal data can more effectively capture the dynamic evolution of employee attitudes and behaviors, thereby enhancing the timeliness and robustness of turnover predictions (Rubenstein et al., 2017; Chen et al., 2011). Particularly for highly mobile occupational groups, long-term tracking data facilitates the identification of both cumulative and stage-specific factors leading to turnover (Hom et al., 2017). Specifically, the operational objectives of this study are as follows: (1) to revise a questionnaire based on established scales and systematically collect data on teacher satisfaction and turnover over a 10-year period; (2) to employ Exploratory Factor Analysis (EFA) to extract core influencing factors from the multidimensional questionnaire and clarify the predictive weight of each dimension on turnover; (3) to construct three machine learning prediction models and compare their performance differences; and (4) to propose actionable teacher retention intervention strategies by integrating the model results with qualitative analysis.

By integrating theoretical frameworks from psychology and organizational management, this study not only addresses the limitations of existing turnover prediction models but also offers practical strategies for human resource management in private universities. The principal innovations of this work are threefold: First, it introduces, for the first time, a 10-year longitudinal dataset focused on faculty in private universities, thereby enhancing the timeliness and robustness of the findings. Second, it proposes a hybrid feature extraction method based on factor analysis, overcoming the feature selection constraints inherent in traditional models, and further validates the effectiveness of the EFA framework through qualitative analysis. Third, it innovatively integrates teachers’ psychological characteristics with organizational management factors, and via a comparative analysis of multiple machine learning models, demonstrates the superiority of the KNN model in predicting faculty turnover. Moving beyond the reliance of conventional models solely on objective data, this research provides valuable guidance for developing teacher retention strategies in private universities, bearing significant theoretical and practical implications.

## 2 Literature review

### 2.1 Theoretical foundations of teacher turnover research

Job satisfaction among teachers is a multidimensional concept influenced by various psychological, organizational, and environmental factors. Herzberg’s Two-Factor Theory posits that job satisfaction is driven by two types of factors: hygiene factors (such as salary and working conditions) and motivators (such as recognition and career development) (Herzberg, 1966). Hygiene factors prevent employee dissatisfaction, while motivators actively enhance satisfaction and loyalty. In the context of higher education, studies suggest that compared to base salary levels, faculty satisfaction in private universities is more sensitive to motivational factors such as research autonomy and career development opportunities (Ali and Botha, 2021). This theory supports the inclusion of factors like “career development” and “administrative support” in satisfaction analyses. Maslow’s Hierarchy of Needs further emphasizes the hierarchical nature of human motivation, indicating that unmet lower-level needs (e.g., job security) can hinder the pursuit of higher-level needs (e.g., self-actualization through teaching or research) (Maslow, 1943). For faculty in private institutions, job stability (a security need) often conflicts with academic freedom (a self-actualization need). When universities fail to balance these priorities, unique tensions arise that may lead to faculty turnover (Kim and Lee, 2020). The Price-Mueller model integrates environmental, individual, and organizational variables to predict turnover behavior (Price and Mueller, 1986). This model identifies job satisfaction and organizational commitment as key mediating factors between external opportunities (e.g., alternative employment options) and turnover decisions. Research shows that even in an unfavorable external job market, dissatisfaction with workload or administrative policies can significantly increase teachers’ turnover intention (Chen et al., 2022).

### 2.2 Applications and research advances of machine learning in turnover prediction

In recent years, machine learning (ML) methods have been widely applied in employee turnover prediction research due to their powerful data-driven and pattern recognition capabilities (Akasheh et al., 2024). As a core branch of artificial intelligence, machine learning can automatically learn patterns from historical data and build predictive models, demonstrating particular strength in handling high-dimensional features and non-linear relationships (Hastie et al., 2009). Based on the availability of data labels and learning objectives, machine learning can be categorized into four main types: supervised learning, unsupervised learning, semi-supervised learning, and reinforcement learning. In the field of employee turnover prediction, supervised learning algorithms such as K-Nearest Neighbors (KNN), Naive Bayes (NB), and Neural Networks (NN) have been shown to be highly effective. For instance, Lazzari et al. (2022) employed seven classifiers, including k-nearest neighbors and decision trees, to analyze multinational survey data and construct a model of employee turnover intention. Their findings indicated that logistic regression and LightGBM performed particularly well in predicting employees’ turnover intention. Park et al. (2024) utilized survey data on career mobility among university graduates from the Korean Employment Information Service, applying logistic regression (LR), K-nearest neighbors (KNN), and Extreme Gradient Boosting (XGB) classifiers for modeling. Their study identified job security as the most significant predictor. Notably, Yiğit and Shourabizadeh (2017) compared the performance of multiple classifiers in predicting employee churn and found that Support Vector Machine (SVM) achieved the best results in terms of accuracy, precision, and F1-score. Fallucchi et al. (2020) validated the advantage of Gaussian Naive Bayes in recall using the IBM dataset, while Al-Darraji et al. (2021) adopted a deep neural network model, achieving an accuracy of 89.11% in employee turnover prediction.

Specifically focusing on the three algorithms examined in this study:

(1) K-Nearest Neighbors (KNN) is an instance-based lazy learning algorithm that classifies data by measuring the distance between different feature values. It is suitable for small-scale datasets and is insensitive to outliers (Cover and Hart, 1967).
(2) Naive Bayes (NB) is based on Bayes’ theorem and assumes feature independence. It is simple, efficient, and well-suited for processing high-dimensional data (Jadhav and Channe, 2016).
(3) Backpropagation Neural Network (BPNN) is a multi-layer feedforward neural network that adjusts weights through the error backpropagation algorithm, exhibiting strong non-linear fitting capability (Rumelhart et al., 1986).

These algorithms have been proven effective in multiple turnover prediction studies.

### 2.3 Research review

The organizational environment is a critical factor influencing faculty turnover. A positive school climate, adequate resources, and supportive administrative management can significantly reduce teacher attrition rates. Conversely, chaotic management, insufficient resources, and stringent accountability systems contribute to teacher dissatisfaction and turnover (Jackson, 2012). Research within the Chinese context indicates that urban living cost pressures, blocked promotion pathways, and a lack of organizational cultural identity constitute three major triggers of faculty attrition in private institutions (Yang and Lu, 2024). Based on survey data from 1,161 teachers in private universities, Chen et al. (2023) found that nearly one-third of the respondents reported turnover intention, with doctoral-degree holders exhibiting the strongest inclination to leave.

However, existing studies utilizing machine learning for predicting faculty turnover have predominantly focused on corporate employees and relied heavily on objective data such as salary levels. These studies often overlook psychological characteristics specific to teachers—such as job satisfaction and professional efficacy—and fail to provide feature importance rankings. As a result, they offer limited practical guidance for developing targeted teacher retention strategies in educational settings (Kim and Lee, 2020). Furthermore, few existing models integrate Exploratory Factor Analysis (EFA) with machine learning methods, thereby inadequately addressing the multicollinearity issues inherent in psychological characteristics (Holton et al., 2022).

To address these limitations, this study proposes a hybrid predictive framework that combines EFA and machine learning. First, EFA is employed to extract latent influencing factors from multidimensional satisfaction questionnaires, effectively mitigating feature redundancy. Subsequently, comparative models are constructed using K-Nearest Neighbors (KNN), Naive Bayes (NB), and Backpropagation Neural Networks to validate the advantages of nonlinear algorithms in predicting faculty turnover. This approach not only enhances the theoretical depth of turnover prediction research but also promotes practical innovation in the field.

## 3 Research object and methods

### 3.1 Research objects

This study focuses on a private university in Western China, referred to as University A, as the empirical research site. University A is one of the first private universities approved by the Ministry of Education. As of December 2023, the university enrolls approximately 20,000 full-time undergraduate students and holds complete academic qualifications. It enjoys a strong social reputation, with no significant public controversies or regulatory penalties during its operation.

The study adopts a longitudinal tracking design, with data collection spanning from January 2015 to December 2024. As shown in Table 1, over the past decade, University A has recruited 497 young and middle-aged faculty members aged between 26 and 45. Of these, 291 teachers (58.55%) remain, while 206 teachers (41.45%) have left. Personnel records were exported from the university’s human resources management system, and data cleaning was performed using a double-blind entry method. Ethical approval for the study was obtained from the Academic Committee of University A. All participants were informed about the survey, provided informed consent, and were assured of the confidentiality and anonymity of their data. In addition, this study commits to providing feedback on the overall research findings to participants after the completion of the study. Specific methods include disseminating results via university email systems and organizing dedicated results-briefing sessions, ensuring that no personal data is involved during the feedback process. These measures further protect participants’ right to information and privacy.

**Table 1:** Data on new hires and departures from university a in the past decade.

Year	New hires in the year	Cumulative new hires	Departures in the year	Cumulative departures
2015	37	37	13	13
2016	48	85	16	29
2017	62	147	21	50
2018	81	228	30	80
2019	70	298	42	122
2020	30	328	9	131
2021	21	349	15	146
2022	32	381	18	164
2023	51	432	22	186
2024	65	497	20	206

### 3.2 Research methods

The study designed the “Teacher Satisfaction Survey for Young and Middle-Aged Faculty in Private Universities,” proposing a feature selection method based on factor analysis and identifying four key feature variables. Subsequently, three machine learning models were used to fit the teacher turnover prediction. The models’ prediction performance was evaluated and compared using metrics such as accuracy, F1 score, and AUC value.

#### 3.2.1 Questionnaire survey method

This study was based on the core dimensions of the short-form Minnesota Satisfaction Questionnaire (MSQ) (Weiss et al., 1967), which contains 20 items, and the Job Satisfaction Survey (JSS) (Spector, 1985). The questionnaire was adapted to align with the organizational characteristics of private universities. The resulting “Teacher Satisfaction Survey for Young and Middle-Aged Faculty in Private Universities” was developed (see Table 2). The survey used a Likert 5-point scale and consisted of 21 items. The Cronbach’s α coefficient for the questionnaire was 0.92, indicating strong internal consistency, with a content validity of 0.91. Additionally, the item discrimination was found to be satisfactory.

**Table 2:** Teacher satisfaction survey for young and middle-aged faculty in private universities.

No.	Items
1	I am satisfied with my current salary and benefits.
2	My recognition of other welfare benefits (endowment insurance, medical insurance, unemployment insurance, work-related injury insurance, maternity insurance, and housing provident fund) provided by the school.
3	My recognition of the title appraisal and appointment system.
4	The frequency of my participation in further education and training.
5	My judgment on my own promotion opportunities.
6	The school supports going out for further academic qualifications (e.g., pursuing a doctoral degree).
7	The school supports going out for temporary on—job training.
8	I can gain a sense of achievement from my current job.
9	I think my current job has a certain degree of challenge.
10	I think my current job is relatively stable.
11	I think my current job is cumbersome and busy. (Reverse—scored)
12	I think my current job can give full play to my professional expertise.
13	The salary and related welfare benefits do not meet my expectations. (Reverse—scored)
14	I am satisfied with the school’s teaching plan arrangement.
15	I am satisfied with the school’s leadership and management style.
16	I agree with the school’s educational and development concepts.
17	I think the school’s assessment and merit-selection methods are scientific and reasonable.
18	My satisfaction with the working conditions (offices, dormitories, laboratories).
19	My satisfaction with the school’s cultural atmosphere.
20	My satisfaction with the relationship between leaders and colleagues.
21	My satisfaction with the relationship with students.

#### 3.2.2 Questionnaire distribution and recovery

This study utilized the university’s human resource management system to conduct an annual tracking survey of faculty satisfaction each December. To ensure a fair comparison of satisfaction levels between teachers who left and those who remained, the analysis uniformly used the most recent questionnaire results obtained during each teacher’s period of employment. Specifically: (1) for teachers who had left, data from their most recent annual survey before departure were used; (2) for teachers still employed, data from the latest survey (December 2024) were used. This approach ensured that all satisfaction data were sourced from the most recent available point in each teacher’s career, effectively capturing their latest state either prior to leaving or while currently employed. This method effectively mitigates measurement errors that could arise from differences in career stages or recent events. Following the above screening and data cleaning procedures, a total of 428 valid questionnaires were included in the final analysis, comprising 227 from currently employed teachers and 201 from those who had left. The distribution of the surveyed teachers by gender, age, educational background, and professional title is presented in Table 3.

**Table 3:** Basic information of the survey.

Name	Option	Frequency	Percentage (%)	Cumulative Percentage (%)
Sex	Male	113	26.4	26.4
	Female	315	73.6	100
	intersex	0	0	0
Age	26–30	123	28.74	28.74
	31–35	107	25	53.74
	36–40	99	23.13	76.87
	41–45	99	23.13	100
Educational Background	Junior College	1	0.23	0.23
	Undergraduate	35	8.18	8.41
	Master’s Degree	345	80.61	89.02
	Doctoral Degree	47	10.98	100
Professional Title	No Professional Title	64	14.95	14.95
	Primary	120	28.04	42.99
	Intermediate	172	40.19	83.18
	Associate Senior	61	14.25	97.43
	Senior	10	2.34	99.77
	Others	1	0.23	100

In the “Satisfaction Survey for Young and Middle-aged Faculty in Private Universities” employed in this study, Item 11 (“I find my current work tedious and busy”) and Item 13 (“My compensation and benefits fail to meet expectations”) were designed as reverse-scored items. This design aimed to control for response bias and avoid habitual responding from participants. The scoring method for the scale was as follows: As the study focuses on turnover prediction, positively worded items were scored as: “Strongly Disagree” = 5 points, “Disagree” = 4 points, “Neutral” = 3 points, “Agree” = 1 point, “Strongly Agree” = 1 point. Conversely, reverse-scored items were scored as: “Strongly Disagree” = 1 point, “Disagree” = 2 points, “Neutral” = 3 points, “Agree” = 4 points, “Strongly Agree” = 5 points. This scoring scheme ensures that the direction of scoring is consistent with the conceptual meaning of the measured dimensions across all items.

#### 3.2.3 Feature extraction method

To address the high-dimensional characteristics and potential information redundancy of the “Teacher Stability Survey for Young and Middle-Aged Faculty in Private Universities,” this study proposes a hybrid feature optimization framework based on factor analysis (Wang et al., 2024). Using EFA for dimensionality reduction, key information from the data can be preserved while reducing its dimensionality. This approach decreases computational complexity and helps classifiers perform classification and prediction more accurately, thereby assessing employee turnover.

Suppose there are n samples and p indicators. Let the random vector $X={\left(X_{1},X_{2},\cdots ,X_{p}\right)}^{T}$, and the common factors to be found be $f={\left(f_{1},f_{2},\cdots ,f_{m}\right)}^{T}$. Then the calculation model is shown in Equation 1.


$$\left\{ \begin{array}{c} x_{1}=u_{1}+a_{11}f_{1}+a_{12}f_{2}+\cdots +a_{1m}f_{m}+\varepsilon_{1}\\ x_{2}=u_{2}+a_{21}f_{1}+a_{22}f_{2}+\cdots +a_{2m}f_{m}+\varepsilon_{2}\\ \vdots \\ x_{p}=u_{p}+a_{p1}f_{1}+a_{p2}f_{2}+\cdots +a_{\mathrm{pm}}f_{m}+\varepsilon_{p} \end{array}\right.$$ (1)

It can also be expressed as x = u + Af + *ε*, where f is the common factor vector, ε is the specific factor vector, and a_ij_ is the factor loading matrix.

The degree to which the original variable x_i_ depends on the common factors is expressed as Equations 2 and 3:


$$h_{i}^{2}=\sum_{j=1}^{m}a_{\mathrm{ij}}^{2}$$ (2)

$$\mathrm{Var}(x_{i})=h_{i}^{2}+\varepsilon_{i}^{2}(i=1,2,\cdots ,p)$$ (3)

$h_{i}^{2}$ represents the variance contribution of the common factors to $x_{i}$, also known as the communality. And $\varepsilon_{i}^{2}$ represents the variance contribution of $\varepsilon_{i}^{2}$ to $x_{i}$, also known as the uniqueness. Moreover, $h_{i}^{2}+\varepsilon_{i}^{2}=1$.

The factors obtained from the principal component analysis were used as the extracted features, which were used as independent variables in the subsequent model fitting for turnover prediction.

#### 3.2.4 Prediction models

##### 3.2.4.1 K-Nearest neighbors (KNN)

KNN is a lazy machine learning method that supports incremental learning. It can be used for non-linear classification and can handle complex decision spaces. When the data volume is small but representative, it has a good classification effect. KNN classifies a sample to be classified by calculating the distances between it and the training samples and selecting the KNN (Everitt et al., 2011). When calculating the sample distances, distance calculation methods such as the Manhattan distance, Euclidean distance, and Chebyshev distance can be used, and the Euclidean distance is generally used. The disadvantages of the KNN are the high computational cost and poor interpretability. In the prediction of teachers’ turnover, the KNN algorithm can intuitively reflect the similarity between samples. Its main calculation process is as follows:

For two n-dimensional samples, and $y=(y_{1},y_{2},\cdots ,y_{n})$ the Euclidean distance between them is shown in the following Equation 4:


$$d(x,y)=\sqrt{\sum_{i=1}^{n}{\left(x_{i}-y_{i}\right)}^{2}}$$ (4)

##### 3.2.4.2 Naive Bayes (NB)

Naive Bayes is a classification method based on Bayes’ theorem, assuming that all features are independent of each other (Panda and Patra, 2008). In the prediction of teachers’ turnover, the Naive Bayes algorithm is simple and efficient and is suitable for processing large-scale data sets. However, this algorithm has a strong assumption of feature independence; therefore, the correlation between features needs to be carefully evaluated in practical applications. The calculation process of the Bayes’ Equation is shown in the following Equation 5.


$$d(x,y)=\sqrt{\sum_{i=1}^{n}{\left(x_{i}-y_{i}\right)}^{2}}$$ (5)

P(y∣x) Where is the posterior probability, is the likelihood probability, is the prior probability, and P(x) is the evidence factor.

#### 3.2.5 Backpropagation neural network (BPNN)

A neural network is a computational model that mimics the connection structure of human brain neurons and has a powerful non-linear mapping ability. In principle, first, the feature items are input, that is, the independent variable items are introduced. When using a neural network model, “pseudo features” can be constructed from the feature items. For example, if the inputs are gender, age, salary, years of work, etc., some “pseudo feature items” (i.e., feature items that do not actually exist and are completely constructed by the model and cannot be interpreted) can be constructed in combination with the activation function. Moreover, there can be multiple levels for constructing “pseudo feature items” (i.e., there can be multiple layers of “hidden layer neurons”), and each layer can have multiple neurons. Finally, the prediction item is obtained through calculation using a mathematical optimization algorithm. In the prediction of teachers’ turnover, the neural network can automatically extract complex patterns in the data, thus achieving high-precision prediction (Liu et al., 2015).

The Equation of the neuron model (taking a single neuron as an example) is See Equation 6:


$$y=f(\sum_{i=1}^{n}w_{i}x_{i}+b)$$ (6)

Where *x_i_* is the input, *w_i_* is the corresponding weight, b is the bias, f(·)is the activation function, and y is the output of the neuron.

The forward propagation calculation Equations of a multilayer neural network are as follows (where x is the input layer, h is the hidden layer, and y is the output layer):

The Equation for the hidden layer can be seen in Equation 7.


$$h_{j}=f(\sum_{i=1}^{n}w_{\mathrm{ij}}^{\left(1\right)}x_{i}+b_{j}^{\left(1\right)}),j=1,2,\cdots ,m$$ (7)

where m is the number of hidden layer neurons.

The Equation for the Output layer can be seen in Equation 8.


$$y_{k}=f(\sum_{j=1}^{m}w_{\mathrm{jk}}^{\left(2\right)}h_{j}+b_{k}^{\left(2\right)}),k=1,2,\cdots ,l$$ (8)

Where l is the number of output layer neurons.

#### 3.2.6 Evaluation metrics

Commonly used evaluation indicators for machine—learning classification models include accuracy (Accuracy, ACC), ROC curve (Receiver Operating Characteristic Curve), F1 score, etc. (Ramakrishnan et al., 2024).

##### 3.2.6.1 Accuracy

According to Equation 9, accuracy refers to the proportion of the number of correctly classified samples to the total number of samples. It intuitively reflects the correctness of the model’s overall classification of the samples.


$$\text{Accuracy}=\frac{\mathrm{TP}+\mathrm{TN}}{\mathrm{TP}+\mathrm{TN}+\mathrm{FP}+\mathrm{FN}}$$ (9)

where TP (True Positive) represents the number of true positives, that is, the number of samples that are actually positive and are predicted as positive by the model; TN (True Negative) represents the number of true negatives, that is, the number of samples that are actually negative and are predicted as negative by the model; FP (False Positive) represents the number of false positives, that is, the number of samples that are actually negative but are predicted as positive by the model; FN (False Negative) represents the number of false negatives, that is, the number of samples that are actually positive but are predicted as negative by the model.

##### 3.2.6.2 F1 Score

The F1 score is an indicator to measure the performance of a classification model, the details are presented in Equation 10. It is the harmonic mean of precision and recall. The maximum value is 1, and the minimum value is 0. It comprehensively considers the accuracy and completeness of the model, and a higher value indicates better overall performance. Among them, precision represents the proportion of actually positive samples among the samples predicted as positive, and recall represents the proportion of samples that are actually positive and are correctly predicted as positive.


$$F1=\frac{2\times \text{Precision}\times \text{Recall}}{\text{Precision}+\text{Recall}}$$ (10)

##### 3.2.6.3 ROC curve (receiver operating characteristic curve)

The ROC curve is plotted with the False Positive Rate (FPR) on the x-axis and the True Positive Rate (TPR) on the y-axis. This shows the model’s ability to distinguish between positive and negative examples under different classification thresholds. The True Positive Rate (TPR) represents the proportion of actually positive samples that are correctly predicted as positive. The Equation for TPR is refer to Equation 11:


$$\mathrm{TPR}=\frac{\mathrm{TP}}{\mathrm{TP}+\mathrm{FN}}$$ (11)

The False Positive Rate (FPR) represents the proportion of actually negative samples that are incorrectly predicted as positive. The Equation for FPR is refer to Equation 12:


$$\mathrm{FPR}=\frac{\mathrm{FP}}{\mathrm{FP}+\mathrm{TN}}$$ (12)

The AUC (Area Under the Curve) is the area under the ROC curve, and its value ranges from 0.5 to 1. The larger the AUC, the better the classification performance of the model.

## 4 Results and discussion

### 4.1 Feature extraction

#### 4.1.1 Factor analysis

This section examines the information compression of the questionnaire data for young and middle-aged faculty in private universities. First, the suitability of the factor analysis for the standardized scores of each sample was tested, including the KMO test and Bartlett’s sphericity test. The results are as follows: (1) The KMO sampling adequacy statistic value is 0.93, indicating a high degree of information overlap and strong correlations between the variables. (2) The Bartlett test yielded a chi-square value of 7855.43, with a significant *p*-value less than 0.05, indicating that the correlations between the variables are significant. Both the KMO and Bartlett’s sphericity tests confirm that the sample data are suitable for factor analysis.

Using the maximum variance rotation method, the variance explained by the first four factors is presented in Table 4. After rotation, the first four factors had eigenvalues greater than 1, and the cumulative variance explained reached 75.59%. Thus, the first four factors were extracted, which successfully condensed the information from the stability survey for young and middle-aged faculty. The variance explained by factor 1 after rotation was 25.41%, which was higher than the variance explained by the other factors, indicating that factor 1 is the most important factor influencing teacher turnover.

**Table 4:** Variance explanation rates of the factors.

No.	Variance Explanation Rate before Rotation			Variance Explanation Rate after Rotation		
	Eigenvalue	Variance Explanation Rate (%)	Cumulative (%)	Eigenvalue	Variance Explanation Rate (%)	Cumulative (%)
1.	10.42	49.61	49.61	5.34	25.41	25.41
2.	2.96	14.09	63.71	3.74	17.80	43.20
3.	1.47	7.01	70.72	3.65	17.37	60.58
4.	1.02	4.87	75.59	3.15	15.02	75.59

Table 5 presents the rotated factor loading coefficients. The results indicate that the communality value for each item exceeded 0.4, signifying a strong association between the items and the factors, and confirming that the factors effectively extract meaningful information. Based on the rotated solution, Factor 1, which primarily loads on items related to salary, social security and housing fund, post-appointment training, and academic credential advancement, was labelled “Compensation, Benefits, and Development.” Factor 2, characterized by high loadings on items concerning sense of achievement, job challenge, and utilization of professional expertise, was named “Professional Efficacy.” Factor 3, which is predominantly associated with school administration, performance evaluation systems, and teaching schedule arrangements, was designated the “School Management” factor. Finally, Factor 4, mainly relating to office facilities and interpersonal environment, was labelled “Work Environment.”

**Table 5:** Factor loading coefficients after rotation.

Items	Factor Loading Coefficients				Communality
	Factor 1	Factor 2	Factor 3	Factor 4	
I am satisfied with the school’s teaching plan arrangement.	0.317	0.359	0.220	0.694	0.759
I am satisfied with the school’s leadership and management style.	0.170	0.398	0.130	0.800	0.844
I agree with the school’s educational and development concepts.	0.301	0.301	0.075	0.767	0.845
I think the school’s assessment and merit-selection methods are scientific and reasonable.	0.056	0.267	0.130	0.814	0.753
I can gain a sense of achievement from my current job.	0.244	0.716	0.251	0.201	0.675
I think my current job has a certain degree of challenge.	0.158	0.817	0.138	0.246	0.772
I think my current job is relatively stable.	0.253	0.813	0.090	0.220	0.782
I think my current job is cumbersome and busy.	0.123	0.766	0.059	0.331	0.715
I think my current job can give full play to my professional expertise.	0.200	0.742	0.145	0.351	0.735
My satisfaction with the working conditions (offices, dormitories, laboratories).	0.386	0.128	0.695	0.155	0.673
My satisfaction with the school’s cultural atmosphere.	0.261	0.086	0.829	0.151	0.786
My satisfaction with the relationship between leaders and colleagues.	0.301	0.185	0.834	0.052	0.823
My satisfaction with the relationship with the students.	0.270	0.154	0.823	0.129	0.791
I am satisfied with my current salary and benefits.	0.856	0.141	0.226	0.128	0.820
My recognition of other welfare benefits (Five Insurances and One Fund) provided by the school.	0.848	0.113	0.214	0.105	0.789
My recognition of the title appraisal and appointment system.	0.783	0.212	0.239	0.148	0.737
The frequency of my participation in further education and training.	0.814	0.191	0.196	0.191	0.774
My judgment on my own promotion opportunities.	0.733	0.186	0.313	0.055	0.673
The school supports going out for further academic qualifications (pursuing a doctoral degree).	0.778	0.167	0.182	0.157	0.691
The school supports going out for temporary on the job training.	0.786	0.230	0.269	0.202	0.784

Through dimensionality reduction via factor analysis, redundant features were effectively eliminated, allowing the model to focus on the most discriminative factors (F1: Compensation, Benefits, and Development; F2: Professional Efficacy; F3: School Management; F4: Work Environment). This outcome aligns with Herzberg’s Two-Factor Theory, wherein both hygiene factors (e.g., salary, work environment) and motivators (e.g., career development, sense of efficacy) collectively influence faculty turnover.

#### 4.1.2 Textual analysis

Based on the dimensional framework derived from the EFA, this study conducted a systematic coding analysis of the textual data from the open-ended question “Other suggestions for enhancing the job satisfaction of young and middle-aged teachers” in the questionnaire. Two independent coders were selected to perform the coding task. Both coders had backgrounds in higher education research (one doctoral candidate and one lecturer). They received unified training prior to coding. Inter-rater reliability was assessed using Cohen’s Kappa coefficient (*κ* = 0.82, *p* < 0.001), indicating high reliability of the coding results. The final coding results are presented in Table 6.

**Table 6:** Text coding multiple response table.

Dimension	Response frequency (*n*)	Response rate	Main topics covered
F1 welfare and development	56	48.7%	Improving salary levels, social insurance, retirement benefits, staffing systems, etc.
F2 career effectiveness	14	12.2%	Research and project support
F3 work environment	16	13.9%	Long commute, housing, dormitory, etc.
F4 school management	29	25.2%	Rigid quantitative assessment system, pressure from “non-promotion, non-retention” policy, excessive administrative workload

As shown in Table 6, the dimension of welfare benefits and career development (F1) received the highest level of attention (*n* = 56, 48.7%). Core appeals focused on optimizing the compensation system (frequency 28), improving social security benefits (frequency 18), and reforming the establishment system (frequency 10), among other aspects. Representative excerpts included:

The school management dimension (F3) ranked second (*n* = 29, 25.2%). Respondents primarily suggested improvements regarding the rigidity of the quantitative assessment mechanism (frequency 15), the pressure from the “up-or-out” policy (frequency 9), and the excessive burden of administrative tasks (frequency 8). Representative excerpts included:

The occupational efficacy dimension (F2) garnered 14 suggestions (12.2%), emphasizing investment in disciplinary construction and the allocation of research resources. A representative excerpt included:

"The basic salary leaves little remains after deducting rent and living expenses. We hope the school can raise the salary standard and adjust the social security and housing fund contribution base to the actual income level. The gap in benefits compared to public institutions is simply too large."

"We, teachers in private institutions, lack the status of public institutional establishment, and there is no security for post-retirement benefits. If the school could promote reform of the establishment system or supplement commercial pension insurance, it would certainly help retain personnel."

The school management dimension (F3) ranked second (*n* = 29, 25.2%). Respondents primarily suggested improvements regarding the rigidity of the quantitative assessment mechanism (frequency 15), the pressure from the "up-or-out" policy (frequency 9), and the excessive burden of administrative tasks (frequency 8). Representative excerpts included:

"The school's evaluation overemphasizes teaching hours and paper counts. Even with excellent teaching outcomes, promotion is impossible without sufficient research points. This 'quantification-only' standard is unsuitable for humanities teachers."

"Filling out five or six administrative forms weekly and attending various irrelevant meetings leaves less than half the time for actual lesson preparation and research. We hope to streamline administrative procedures and return energy to teaching and research."

The occupational efficacy dimension (F2) garnered 14 suggestions (12.2%), emphasizing investment in disciplinary construction and the allocation of research resources. A representative excerpt included:

“The school’s laboratory equipment is outdated, and the process for applying research funding is cumbersome. There are simply no conditions for conducting innovative research. It would be helpful to increase seed funding for research and equipment investment.”

The work environment dimension (F4) received 16 suggestions (13.9%), primarily focusing on practical needs such as housing security (frequency 5) and commuting convenience (frequency 3). A representative excerpt included:

“The school is located too far from the city center, surrounding rentals are expensive and inconvenient. We hope the school can provide faculty transitional housing or housing subsidies.”

Qualitative analysis further revealed that the F1-F4 dimensions covered 92.4% (*n* = 115) of all suggestions, and their internal item factor loadings were all >0.65, confirming the effectiveness of the EFA model. The results also provide empirical evidence for private university administrators to build a multidimensional retention mechanism.

### 4.2 Model prediction results

The factors obtained from the factor analysis (F1 Welfare and Development, F2 Career Effectiveness, F3 School Management, F4 Work Environment) were used as independent variables, with employee turnover (whether the employee left or not) as the dependent variable. The training set proportion was set to 0.8, and three models—K-Nearest Neighbors (KNN), Naive Bayes (NB), and Backpropagation (BPNN) neural network—were used for modeling. The main parameter settings for each machine learning model are shown in Table 7.

**Table 7:** Main parameter settings of the models.

Model	Parameter Settings
KNN	Number of K—nearest neighbors: 5 Sample voting rights: Equal—ratio voting rights Neighbor search method: Automatic mode Distance calculation method: Euclidean distance
NB	Feature distribution: Gaussian distribution Smoothing parameter (alpha value): 1.0
BPNN	Architecture: Multi—Layer Perceptron (MLP) with 1 hidden layer Number of neurons in the hidden layer: 100 Activation function: ReLU (Rectified Linear Unit) function Weight optimization method: Stochastic Gradient Descent (SGD) Regularization: L2 regularization is introduced with a regularization coefficient of Maximum number of iterations: 200 Optimization tolerance (based on the loss function value):

#### 4.2.1 Model performance comparison

The prediction results of the models on the training and test sets are shown in Table 8. On the training set, both KNN and the BPNN demonstrated the highest classification capability, with accuracies of 84.21 and 83.04%, respectively. Their recall rates both exceeded 85%, indicating that these two types of models can effectively identify positive samples within the target category. On the test set, although NB had the lowest accuracy on the training set (77.19%), it exhibited generalization ability comparable to the best model on the independent test set, with an accuracy of 83.72% and an F1-score of 84.09%.

**Table 8:** Model prediction results for the training and test sets.

Algorithm model	Accuracy		Precision		Recall		f1-score	
	Training set	Test set	Training set	Test set	Training set	Test set	Training set	Test set
KNN	83.64%	84.21%	79.49%	79.38%	89.42%	91.67%	84.16%	85.08%
NB	77.19%	83.72%	73.20%	77.08%	84.52%	92.50%	78.45%	84.09%
BPNN	82.94%	83.04%	78.72%	78.95%	88.94%	89.29%	83.52%	83.80%

Table 9 provides the prediction results of various models for the overall training and testing datasets. All models showed accuracy rates ranging from 79.05 to 83.64% on the overall dataset, with KNN achieving the best performance, with an overall accuracy rate of 83.64% and an F1 score of 84.16%.

**Table 9:** Prediction results of multiple models for the overall data of the training and test sets.

Algorithm model	Accuracy	Precision	Recall	F1 score
KNN	83.64%	79.49%	89.42%	84.16%
NB	77.34%	71.76%	87.98%	79.05%
BPNN	82.94%	78.72%	88.94%	83.52%

#### 4.2.2 ROC curve validation

ROC curves were constructed for KNN_Prediction_probability, NB_Prediction_probability, and BP_Prediction_probability, with the three models showing high diagnostic value for turnover prediction. As shown in Table 10, all models demonstrated strong discriminative ability, with the Area Under the Curve (AUC) values all exceeding 0.80, indicating high prediction accuracy. Specifically, the KNN model had the highest AUC value of 0.901 (95% confidence interval: 0.872 ~ 0.930), followed by the BPNN (AUC = 0.888; 95% confidence interval: 0.855 ~ 0.921).

**Table 10:** ROC results AUC summary.

Items	AUC	Standard error	p	95% CI
KNN_Prediction_probability	0.901	0.015	0.000	0.872 ~ 0.930
NB_Prediction_probability	0.855	0.018	0.000	0.819 ~ 0.891
BP_Prediction_probability	0.888	0.017	0.000	0.855 ~ 0.921

**Figure 1:**
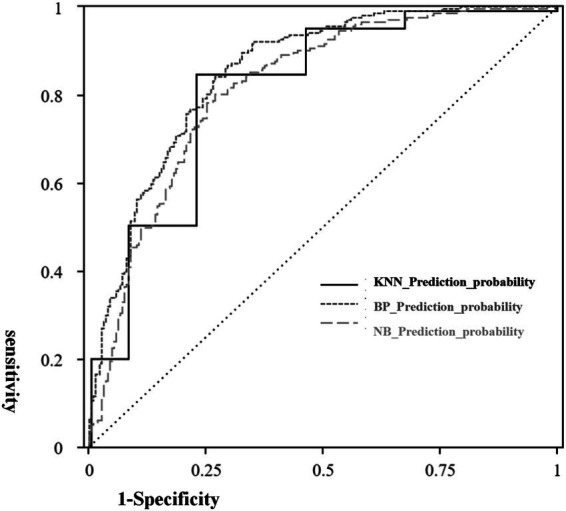
Roc curve.

Figure 1 shows the ROC curves for the three models. The KNN model’s curve is positioned at the topmost, closest to the ideal model point (where sensitivity = 1 and specificity = 1), indicating that it performed the best in distinguishing whether teachers left or not.

## 5 Discussion

This study reveals a significant association between the four dimensions extracted by EFA (welfare benefits and career development, occupational efficacy, work environment, and school management) and teacher turnover behavior, further supporting the applicability of classical organizational behavior theories in the context of private education. For instance, the “welfare benefits and career development” factor, as the dimension with the highest weight, strongly aligns with the fundamental role of “hygiene factors” in Herzberg’s Two-Factor Theory (Herzberg, 1966). This finding is also consistent with conclusions from numerous recent machine learning-based employee turnover prediction studies, where multiple reviews and empirical analyses indicate that salary is one of the most important and stable predictors (Akasheh et al., 2024; Najafi-Zangeneh et al., 2021). Simultaneously, the importance of the “occupational efficacy” factor echoes the persistent influence of higher-level self-actualization needs on career choices within Maslow’s Hierarchy of Needs theory (Maslow, 1943). This result aligns with the findings of Chen et al. (2023) regarding private university teachers, which identified professional development opportunities and work autonomy as key motivational factors affecting turnover intention. Furthermore, Ma’s (2013) empirical research on occupational stress and turnover intention among teachers in private higher vocational colleges indicated that school management, along with four other stress factors—teacher compensation, role responsibilities, personal development, student factors, and interpersonal relationships—significantly influenced turnover intention. This highly overlaps with the four dimensions identified in this study, further confirming the composite nature of turnover drivers among private university teachers. These drivers encompass basic hygiene factors such as compensation and security, as well as motivational factors involving management climate and career growth, fitting the multi-causal and hierarchical patterns revealed by general employee turnover prediction research (Akasheh et al., 2024; Rombaut and Guerry, 2017). This suggests a certain degree of stability and generalizability in the factors influencing turnover among private university teachers across different periods and research contexts.

Regarding model prediction performance: the KNN algorithm demonstrated relatively outstanding results in the overall tests. Particularly on the test set, all four key classification metrics reached relatively high values, with accuracy and F1-score reaching 83.64 and 84.16%, respectively. This indicates that the algorithm effectively captured the data characteristics within this study’s dataset, resulting in favorable predictive performance for teacher turnover. This finding aligns with conclusions from existing studies utilizing machine learning for employee turnover prediction, which suggest that KNN exhibits stable performance when handling small-to-medium-scale classification problems with distinct features (Yiğit and Shourabizadeh, 2017; Lazzari et al., 2022). The neural network model demonstrated strong generalization ability between the training and test sets, highlighting its capability to handle complex nonlinear relationships. By leveraging their powerful capacity for feature combination and mapping, neural networks have shown high discriminative power in several employee retention prediction studies (Al-Darraji et al., 2021; Srivastava and Eacchempati, 2021). However, its high computational complexity and reliance on large-scale data limited its practicality in the context of this study’s limited sample size. In contrast, the Naïve Bayes algorithm achieved the highest recall rate on the test set (92.50%), indicating its unique value in reducing the “risk of missed detection.” This makes it particularly suitable for institutions that prioritize the identification of high-risk teachers. Similarly, Fallucchi et al. (2020) also noted that Naïve Bayes performs excellently in terms of recall rate when processing highly imbalanced human resources data, making it especially apt for the preliminary screening of high-risk populations.

## 6 Conclusion

This study addresses the issue of teacher turnover prediction in private universities in China, using a questionnaire survey method and applying EFA for feature extraction. Three machine learning models were compared based on their ability to predict teacher turnover. The main conclusions are as follows:

The EFA framework helps institutions identify key drivers of turnover. For example, “Welfare and Development” (variance contribution rate: 25.41%) and “Career Effectiveness” (variance contribution rate: 17.80%) are significantly associated with negative evaluations that influence turnover decisions. The “Welfare and Development” factor highlights the importance of material factors, such as salary, training, and promotion opportunities, in teachers’ turnover decisions. The “Career Effectiveness” factor emphasizes the role of psychological factors, such as job satisfaction and work challenges, To a certain extent, it provides a clear direction for colleges and universities to develop targeted teacher retention strategies. Text coding reveals that 48.7% of improvement suggestions focus on issues related to the F1 factor, such as optimizing the salary system and enhancing social security benefits. Qualitative analysis further validates the effectiveness of the EFA framework.

In terms of model prediction, the K-Nearest Neighbors (KNN) algorithm is relatively optimal, with an accuracy of 83.64%, an F1 score of 84.16%, and an ROC curve AUC value of 0.901. A comparison of multiple machine learning models revealed that Naive Bayes (NB), with its high recall rate, is well-suited for initial risk screening, while KNN’s stability makes it ideal for in-depth analysis of key personnel. On the premise that data quality and feature structure are stable, it provides a quantitative analysis tool for human resource management in private colleges and universities.

The “EFA + Machine Learning” hybrid framework constructed in this study provides a reference for conducting turnover prediction in similar institutions. By employing EFA to eliminate redundant features and focus on influential factors, it addresses the issue of multicollinearity in psychological characteristic data. Combining this with multi-model comparative validation overcomes the reliance on a single algorithm common in traditional research. Furthermore, the non-intrusive design of the satisfaction questionnaire reduces teachers’ psychological defensiveness and enhances data authenticity, making this framework more practical in sensitive contexts like private universities where personnel data is highly guarded.

### 6.1 Research limitations

1. Limited sample representativeness: The research data were sourced from only one private undergraduate university in Western China. Consequently, the applicability of the conclusions is primarily limited to private universities similar to the research object in terms of scale and faculty structure. The framework’s adaptability to public universities or private universities in different regions and at different levels requires further validation.
2. Potential bias in data processing: In the data collection process, data from teachers who left was their last survey response before departure, while data for retained teachers came from the most recent survey (December 2024). The time difference and status disparity between these data points may introduce bias.
3. Limitations in variable dimensions: The questionnaire design primarily relied on subjective satisfaction feedback and did not incorporate key objective performance indicators (such as salary records, research output) or external factors like the job market environment.
4. Lack of analysis on interacting factors: The study identified influencing factors within dimensions like “Welfare Benefits and Career Development” and “Occupational Efficacy.” However, constrained by the article’s length, it did not further explore the interaction effects between demographic characteristics (such as gender, professional title, age) and these core influencing factors.

### 6.2 Future research directions

Based on the above limitations, future research could be deepened in the following directions:

(1) Expanding samples and data sources: Future studies could extend to multi-regional institutional comparisons, integrate multi-source data (e.g., salary, research achievements) to build dynamic prediction systems, and explore the application of machine learning models for cross-institutional generalization.
(2) Optimizing data processing and model design: Subsequent research should consider introducing time-series analysis methods to construct dynamic turnover prediction models capable of capturing the evolution of teachers’ turnover intentions.
(3) Deepening the analysis of interacting factors: Research should investigate the interaction mechanisms between demographic dimensions (such as gender, age, education level, professional title) and core factors like “Welfare Benefits” and “Occupational Efficacy.”
(4) Integrating policy and environmental dynamics into the framework: External factors, such as macro-policy changes (e.g., reforms of the faculty establishment system in private universities) and regional disparities in educational resource allocation, should be incorporated into the analysis. Exploring the moderating effects of the institutional environment on teachers’ turnover decisions will make the research conclusions more aligned with the practical development context of private universities.

## Data Availability

The raw data supporting the conclusions of this article will be made available by the authors, without undue reservation.
